# Controlled Continuous Patterning of Spherical Stainless Steel by Multi-Axis Linkage Laser Milling

**DOI:** 10.3390/mi13081338

**Published:** 2022-08-18

**Authors:** He Li, Junjie Zhang, Wenqi Ma, Yuan Liu, Xuesen Zhao, Zhenjiang Hu, Xiaohui Wang, Min Sheng, Tao Sun

**Affiliations:** 1 Center for Precision Engineering, Harbin Institute of Technology, Harbin 150001, China; 2 School of Astronautics, Harbin Institute of Technology, Harbin 150001, China; 3 School of Mechanical Engineering, University of Jinan, Jinan 250022, China; 4 Wuhan Maritime Communication Research Institute, Wuhan 430205, China

**Keywords:** laser surface texturing, non-planar patterning, multi-axis milling, tool path, posture

## Abstract

While laser surface texturing is promising for the fabrication of planar surface microstructures, the continuously patterning with micrometer accuracy of non-planar surface on miniature parts with large curvature by laser ablation is challenging. In the present work, we demonstrate the feasibility of applying the proposed multi-axis laser milling in continuous patterning of 25 mm diameter spherical stainless steel with high uniformity and precision, based on a strategy of simultaneously adjusting the position and the posture of laser-surface interaction point for enabling the constant coincidence of laser beam with ablated surface normal. Specifically, a miniaturized five-axis platform for controlling workpiece motion with high degree-of-freedom is designed and integrated with a fixed nanosecond pulsed laser beam operating at 1064 nm. The precise path of laser-surface interaction point is derived based on the projection and transformation of pre-determined planar pattern on spherical surface. Meanwhile, a virtual prototype of the multi-axis laser milling with embedded interpolation algorithm is established, which enables the generation of NC codes for subsequent laser milling experiments. Furthermore, the sampling of laser processing parameters particularly for spherical surface is carried out. Finally, complex patterns are continuously structured on the spherical surface by employing the proposed multi-axis laser milling method, and subsequent characterization demonstrates both long range uniformity and local high accuracy of the fabricated patterns. Current work provides a feasible method for the continuous laser surface texturing of non-planar surfaces for miniature parts with large curvature.

## 1. Introduction

In recent decades, surface texturing on non-planar surface has been widely applied to enhance surface functionalities of components and parts in fields of tribology, optics, and surface engineering [1,2,3,4,5,6,7]. Along with the high demanding of non-planar surface textures, a variety of corresponding mechanical texturing methods has been proposed, such as computer numerical control (CNC) single-point diamond cutting [8,9,10], grinding [11,12], and milling [13,14], etc. Although above mechanical texturing methods possess high flexibility and high precision, they suffer greatly from the severe tool wear as well as the limitation in material type that can be processed. Therefore, non-contact texturing methods on non-planar surface with high flexibility and high precision are greatly desired.

Laser surface texturing (LST) has been demonstrated to be a non-contact method for fabricating planar surface textures for a variety of materials [15,16]. In the LST, the focusing of high-energy laser beam on workpiece surface leads to dramatic increase of local surface temperature within a short duration, which results in the melting and subsequent evaporation of ablated material. By controlling the movement of focused laser beam under proper laser processing parameters, textures in various shapes, such as groove, microcraters, and micromesh, can be fabricated on planar surface [17,18,19,20,21].

However, the work dealing with the LST on non-planar surface is rather limited, mainly due to the difficulty in the precisely focusing of laser beam on curved surface with existing curvature. Specifically, when focused laser beam is directly irradiated on curved surface, the beam incidence angle (BIA) changes continuously with curved surface position, accompanied with the change of focused spot from a circle to an ellipse. Consequently, the unevenly distributed energy of laser beam on the ablated curved surface results in varied depths and widths of textures, which seriously affect the long-range processing quality of as-fabricated textures. Wang et al. reported that the change in BIA strongly affects the ablation size and ablation depth during laser processing of cylindrical surface textured patterns [22]. Therefore, how to realize the precise focusing of laser beam on curved surface, in particular the constant coincidence of laser beam with the normal of ablated curved surface, is central for achieving uniform ablation quality and high precision textures on non-planar surface.

Recently, the multi-axis laser milling (MALM) method, which integrates the laser beam deflection unit (LBDU) with a multi-axis motion platform, has been proposed to realize the LST on non-planar surface. Specifically, the multi-axis motion platform changes the position and the posture of the workpiece or the laser beam to realize the precise focusing of laser beam on curved surface. Currently, there are two kinds of strategies of MALM proposed for fabricating surface textures on non-planar surface. One strategy is the segmentation algorithm adopted by Cuccolini et al. [23] and Batal et al. [24]. Specifically, the curved surface with large area to be ablated is first triangulated into compositional segmented areas. The posture of laser beam with respect to surface normal is only changed once for each segmented area, which is then subjected to laser ablation by following the fashion of planar surface texturing. The other strategy is the layer slicing algorithm employed by Jiang et al. [25] and Yung et al. [26], especially for the LST on rotationally symmetrical surface. Specifically, the curved symmetrical surface to be ablated is sliced into several parallel girdles, and the posture of laser beam with respect to surface normal is only changed once for each girdle, after that laser ablation of the girdle is performed by following the fashion of planar surface texturing.

Although the aforementioned work demonstrates the feasibility of applying the MALM in the LST on non-planar surfaces, the strategies utilized in previous work mainly lead to discontinuous texturing, rather than continues patterning. Specifically, for both the strategies of segmentation algorithm and layer slicing algorithm, the movements of laser beam between segmented areas or sliced girdles are achieved through the jog of the multi-degree-of-freedom motion platform, thus leading to discontinuous LST accompanied with the formation of clear boundaries between segmented areas or sliced girdles. Cuccolini et al. triangulated the free-form surface into planar fields by using LBDU to process different local fields, and found that there are joining errors between the fields observed [23]. Batal et al. adopted the segmentation algorithm to divide the spherical surface of the titanium alloy into triangular areas for subsequent laser processing, and found that the stitching gaps between neighboring areas are clearly visible [24]. Furthermore, the posture of laser beam with respect to surface normal is unchanged during the laser ablation of individual segmented area or sliced girdle with existing curvature, the resulting varying BIAs with evolution of laser-surface interaction point coordinate generally lead to non-uniform precision of laser ablation. Thus, a strategy of simultaneous adjustment of the position and the posture of laser-surface interaction point is essentially desired for the LST of non-planar surface. In addition, previous work of MALM mainly uses ultrafast picosecond and femtosecond lasers with high accuracy but low efficiency and high cost, and there is no research reported on MALM on non-planar surface using nanosecond laser with moderate accuracy but low cost, high power, and high robustness. It is also necessary to explore the possibility of applying the nanosecond laser in fabricating textures with high quality on non-planar surface.

Therefore, in view of above issues in the MALM for non-planar surface texturing, in this work we propose a novel method of CNC multi-axis linkage laser milling, which is implemented on a five-axis motion platform integrated with nanosecond pulsed laser to achieve the continuous patterning of spherical stainless steel. Specifically, the multi-axis motion platform enables the simultaneous adjusting of both the position and the posture of laser-surface interaction point during laser milling process, which is established to realize the constant coincidence of laser beam with ablated surface normal. In such a way, long-range uniformity and local-range high accuracy of textures can be realized. More importantly, the ablated surface is not needed to be pre-segmented or sliced, and there is no discontinuous boundary formed. The principle of continuous MALM on spherical surface, including the projection algorithm of planar pattern on non-planar surface, is analyzed. Furthermore, the path planning of laser-surface interaction point is designed, and then is validated by a virtual environment of MALM process, which also enables generation of NC codes for subsequent MALM experiments. Finally, different complex continuous patterns are successfully fabricated on the stainless steel spherical surface by employing the proposed MALM strategy, and subsequent characterization demonstrates the continuous transition in neighboring regions of fabricated patterns with high local precision and high long-range uniformity. The proposed method in this work extends the capability of LST on non-planar surfaces.

## 2. Methodology

### 2.1. Experimental Setup of MALM

**Figure 1:**
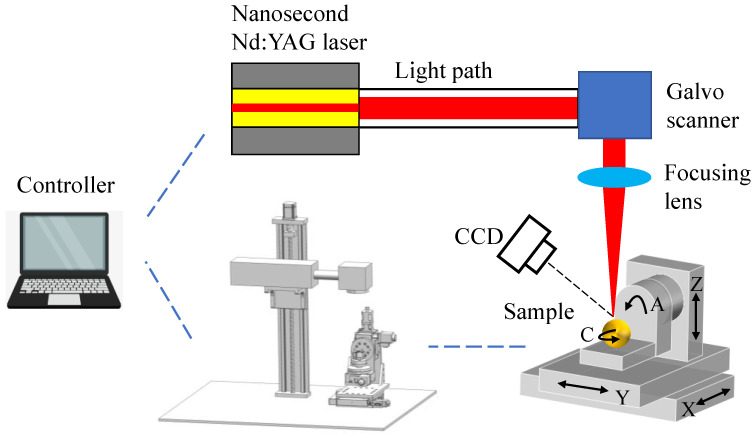
Schematic illustration of the MALM configuration.

Figure 1 presents the schematic illustration of the MALM, which is composed of a desktop computer connected with motion controller, a laser beam system, a CCD camera, and a five-axis motion platform. The pre-setting of laser processing parameters, the focusing of laser beam on ablated surface and the movement of the motion platform are all accomplished by the controller. The laser beam system consists of nanosecond pulsed laser, light path, Galvo scanner, and focusing lens. The nanosecond pulsed laser has a wavelength of 1064 nm, a maximum laser energy of 30 W, and a maximum repetition rate of 200 kHz. The CCD is employed for facilitating the focusing of laser beam on curved surface. The focused spot diameter for a focusing length of 100 mm is 27 μm.

**Figure 2:**
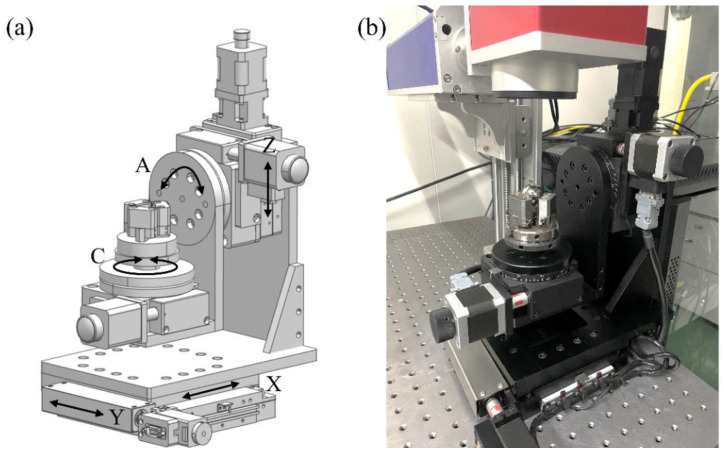
(**a**) Designed and (**b**) realized five-axis motion platform.

The home-made five-axis motion platform is composed of three linear motions of X, Y, and Z axis and two rotational motions of A and C axis, as depicted in Figure 2a. The main specifications of the motion platform are listed in Table 1. The linkage of each axis is realized by the Turbo Clipper motion controller. The workpiece of a spherical 304 stainless steel with a diameter of 25 mm is fixed on the C-axis. Figure 2b presents realized five-axis MALM platform.

Prior to MALM, the X, Y, and Z axes of the motion platform are adjusted to make the unfocused laser beam coincident with the highest point of stainless steel sphere. Then the laser beam is focused on workpiece surface by vertically moving the whole light path including the galvo scanner through an additional vertical guide way. After focusing, in the following MALM process the laser beam is fixed, and the platform drives the workpiece to travel a specific motion path, which is pre-determined according to the projection of designed planar pattern onto a non-planar surface. Subsequently, the virtual simulation of the motion path involved in the MALM process is carried out, which enables the checking of milling process as well as the generation of the NC codes for driving the actual motion of the platform.

### 2.2. Projection Algorithm of Planar Surface Pattern on Non-Planar Surface

In the proposed MALM process, the laser beam is fixed without using the LBDU. The motion of workpiece is purely drived by the five-axis platform following the pre-specificed motion path. Therefore, the projection of the designed planar surface pattern on curved surface is critical for deriving the motion path of workpiece. The motion path of workpiece is decomposed into three linear motions and two rotational motions by a specific projection algorithm. Specifically, the three linear axes X, Y, and Z are used to determine the position of laser-surface interaction point. The two rotational axes A, C are used to adjust the posture of laser-surface interaction point, so that the laser beam always coincides with the ablated surface normal.

**Figure 3:**
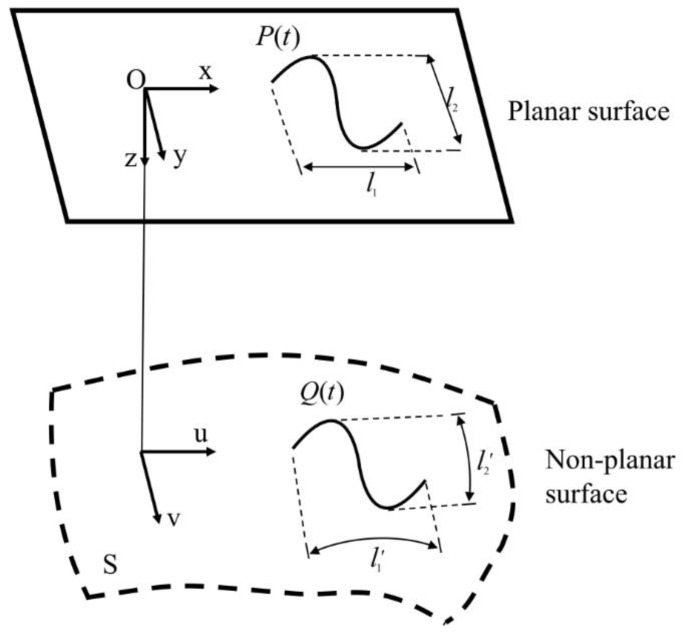
Schematic diagram of equal arc length projection of planar surface curve on non-planar surface.

In this work, the algorithm of equal arc length projection is used for the projection of planar surface pattern onto non-planar surface, as illustrated in Figure 3. Specifically, the curve designed on the two-dimensional plane O-xyz is expressed in the parametric form as *P*(*t*), which contains in-plane *x* and *y* axis, and normal-plane *z*-axis. The as-established Cartesian cooridnate system intersects with the origin of (*u,v*) coordinate system of curved surface S, for which the direction of *x*-axis and *y*-axis is consistent with the direction of u-axis and *v*-axis, respectively. Specifically, “*u*” along the u-curve and “*v*” along the v-curve is determined by measuring the arc length distance along the *x*-axis and the *y*-axis, respectively, i.e., $l_{1}=l_{1}^{\prime },l_{2}=l_{2}^{\prime }$. Consequently, the arc length in planar curve *P*(*t*) projected along the *x*-axis and *y*-axis on the planar surface is equal to the length in non-planar curve *Q*(*t*) projected along the u-axis and v-axis on the non-planar surface, respectively. Therefore, according to the principle of equal arc length projection, the relationship between the planar curve on the plane *P*(*t*) and the projectied non-planar curve on the curved surface *S*(*u*,*v*) is expressed by Equation (1) [27]:

(1) $$\int_{L}P(t)dt=\int S[u(t),v(t)]\sqrt{u^{\prime }{}^{2}(t)+v^{\prime }{}^{2}(t)}dt$$

### 2.3. Digital Simulation of MALM

**Figure 4:**
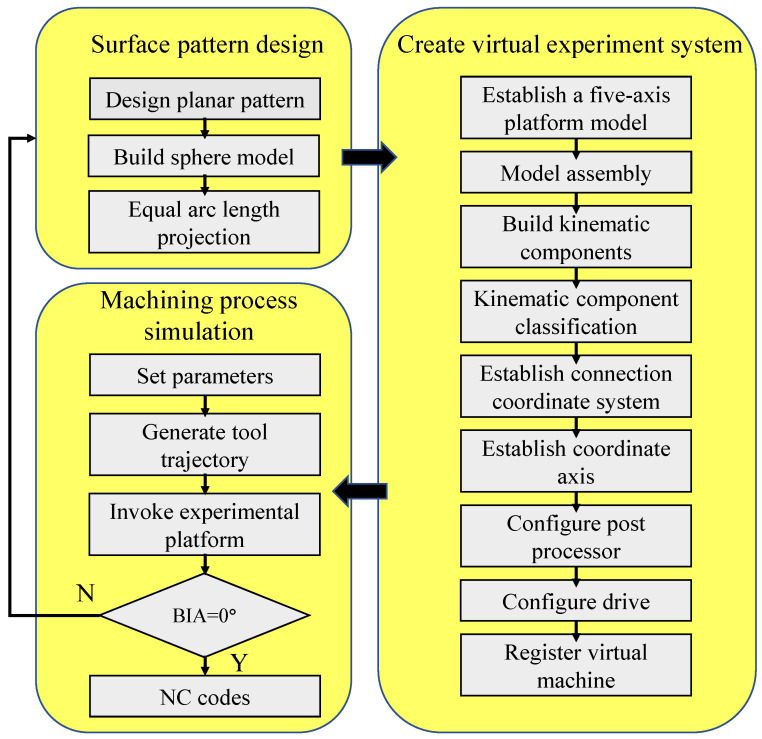
Flowchart of digital simulation of MALM of non-planar surface.

To extract the motion path of workpiece for driving the five-axis motion platform and deriving according to NC codes for driving motions, a virtual machining environment of MALM is established to digitally simulate the laser milling process of non-planar surface. In particular, the configuration of virtual motion platform, including structure, size, motion range, and speed of each axis, is identical to the experimental one, in such a way that the NC codes generated by the digital simulation can be directly used for the experimental machining. The flowchart of the digital simulation of MALM is shown in Figure 4. First, the designed planar surface pattern is projected onto the non-planar surface through the equal arc length projection algorithm to obtain the motion path of workpiece. Subsequently, the assembly model of the five-axis motion platform is established according to the experimental configuration, and the kinematic attributes and coordinate system of each axis are specified according to machine tool kinematic chains. Then, according to the actual parameters and accuracy of the platform, the post processor and driver are configured to establish a complete virtual system for the digital simulation of MALM. Finally, the MALM simulation according to the motion path is carried out within the virtual platform, which also derives corresponding NC codes.

## 3. Results and Discussion

### 3.1. Motion Path Planning of MALM

**Figure 5:**
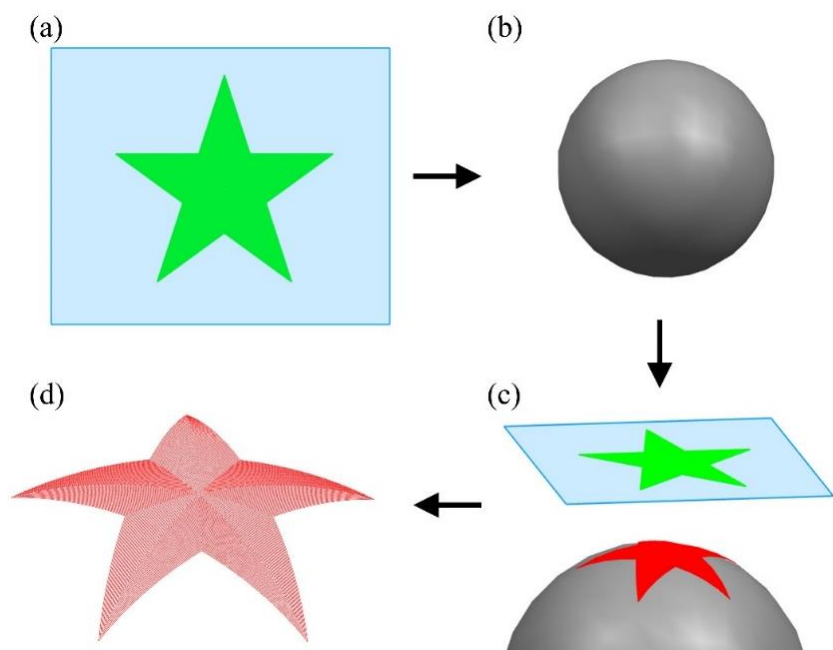
Projection of planar pentagonal pattern onto spherical surface. (**a**) Planar pentagonal pattern; (**b**) spherical surface; (**c**) projection by equal arc length algorithm; (**d**) projected non-planar pentagonal pattern.

In this work, the feasibility of MALM integrated with above algorithm is demonstrated by fabricating a pentagonal pattern on a 304 stainless steel spherical surface. First, the sketch of a regular pentagonal pattern with a circumscribed circle diameter of 15 mm is drawn on a planar surface, as shown in Figure 5a. Then, a sphere with a diameter of 25 mm shown in Figure 5b is introduced for subsequent projection. The planar pentagonal pattern is offset to the sphere center with an interval of 40 μm, enabling the full covering of the pentagonal pattern on the spherical surface. Subsequently, the projection of planar pentagonal pattern on the spherical surface is performed by following the equal arc length projection algorithm, as shown in Figure 5c. Finally, the processing path of the projected non-planar pentagonal pattern on the spherical surface is derived, as shown in Figure 5d.

**Figure 6:**
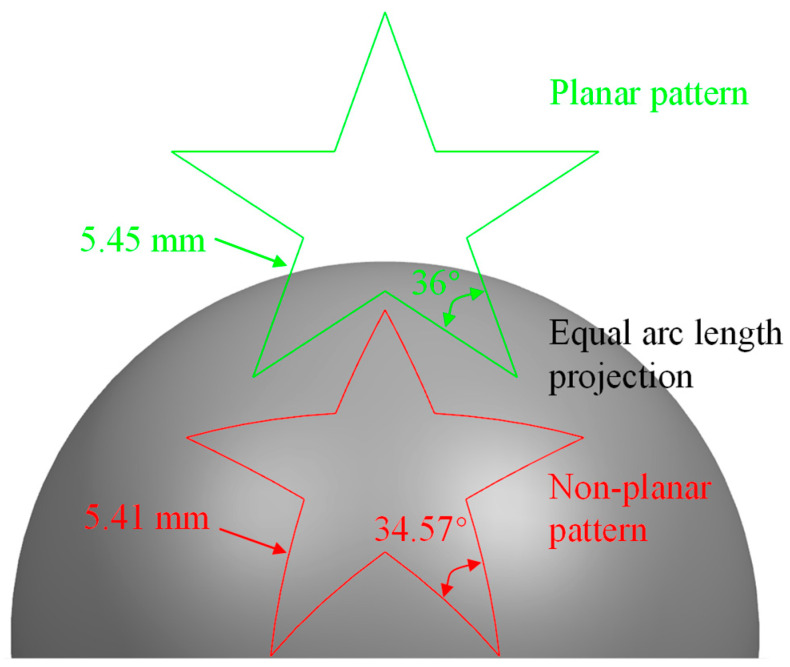
Geometrical parameters of planar pentagonal pattern and projected non-planar pentagonal pattern onto spherical surface.

Figure 6 shows the projection of planar pentagonal pattern with an outer circle diameter of 15 mm onto the spherical surface with a diameter of 25 mm by using the equal arc lenth projection algorithm. The equal arc length projection attaches a plane gragh to the curved surface, which can maximize the size and angle of the projection graph to reduce the structural distortion, as shown in Figure 3. Specifically, the conor angle of 36° and the segment length of 5.45 mm on the planar surface changes to 34.57° and 5.41 mm on the spherical surface, respectively. The small deviations of 4.0% for the conor angle and 1% for the segment length after the projection indicate the ultra-low structural distortion of the projected non-planar pattern, which provides guarantee for achieving high precision for the MALM.

**Figure 7:**
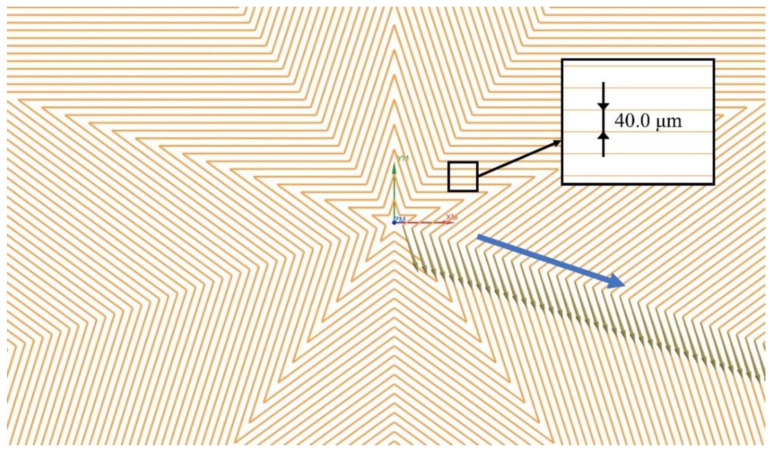
Derived paths of laser-surface interaction points based on the topology of parallel contours of pentagon.

Figure 7 shows the enlarged view of the motion path of projected non-planar pentagonal pattern. The entire motion path adopts a topology of parallel contours of pentagon with an equal spacing of 40 μm. In the MALM, the laser-surface interaction point starts with the innermost ring of pentagonal pattern, and is processed clockwise by tracking a series of laser-surface interaction points along the motion path. After finishing the first pentagon, the laser-surface interaction point moves outwards by a distance of 40 μm to process the next pentagon following the same fashion along the path indicated by the blue arrow, until the processing of the entire designed pattern is completed. To ensure that the laser beam is always perpendicular to the spherical surface, the forward step between the neighboring laser-surface interaction points should be as small as possible. Considering the motion accuracy of the motion platform, a forward step of 15 μm is adopted. Then, the data point of NC codes corresponding to the laser processing path is generated through post-processing, and the NC code file is output for actual MALM experiment.

### 3.2. Sampling of Laser Processing Parameters

Prior to the fabrication of non-planar patterns by MALM, the sampling of laser processing parameters on curved surface is carried out to improve the machined surface quality. Specifically, the influence of laser energy density on the laser ablation quality is evaluated. Five laser energy densities, as 2.62, 3.93, 5.24, 6.55, and 7.86 J/cm^2^, respectively, are considered. For each laser energy density, while the sphere with a diameter of 25 mm is mounted on the moving platform with BIA normal to the spherical surface, the fabrication of groove on the spherical surface with a diameter of 25 mm is achieved by rotating C-axis for one cycle with a speed to 20°/s. The maximum repetition rate of 200 kHz is utilized.

**Figure 8:**
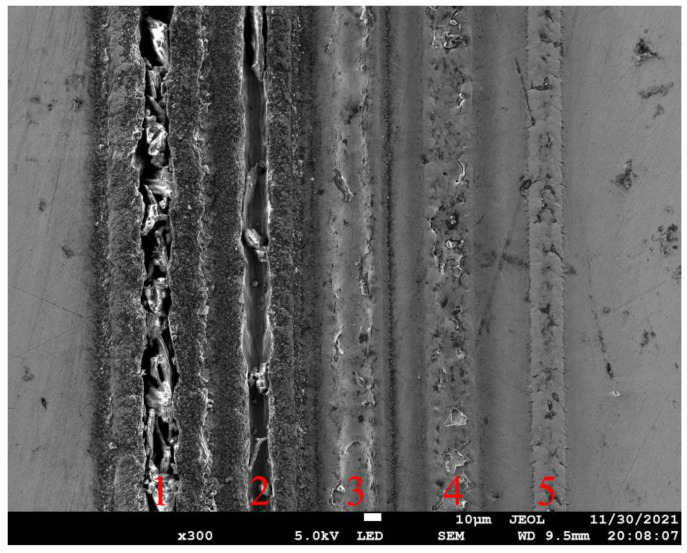
SEM images of ablated grooves on stainless steel sphere under different laser energy densities: 1 for 7.86 J/cm^2^, 2 for 6.55 J/cm^2^, 3 for 5.24 J/cm^2^, 4 for 3.93 J/cm^2^, and 5 for 2.62 J/cm^2^.

Figure 8 presents SEM images of fabricated grooves on spherical surface under different laser energy densities. For the high laser energy densities ranging from 3.93 J/cm^2^ to 7.86 J/cm^2^, there are pronounced cracking and strong heat affected zone (HAZ) formed, accompanied with obvious spatters around the grooves and a recast layer that is melted and solidified. For the low laser energy density of 2.62 J/cm^2^, the ablation groove is significantly regular and uniform with limited recast layers and spatters, and the HAZ size is also small. Therefore, a laser energy density of 2.62 J/cm^2^ is used in subsequent experiments to ensure the high quality of the textures.

**Figure 9:**
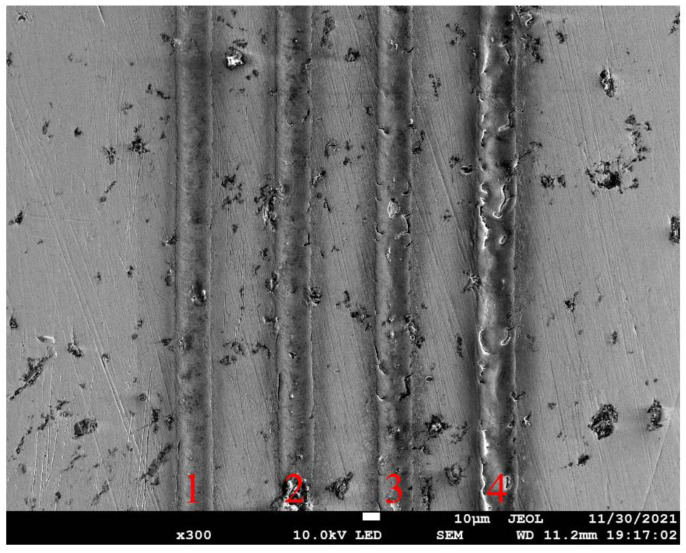
SEM images of ablated grooves on stainless steel sphere under different rotation speeds of C-axis: 1 for 20°/s, 2 for 15°/s, 3 for 10°/s, and 4 for 5°/s.

While the MALM proposed in the present work is mainly achieved by the five-axis motion platform, the scanning speed of fixed laser beam on the workpiece depends on the moving speed of the rotating axis of the platform. Therefore, the influence of rotation speed of C-axis on the laser ablation of groove on spherical surface is investigated. Four rotation speeds of C-axis are considered, as 5, 10, 15, and 20°/s, respectively. For each rotation speed, the laser energy density is 2.62 J/cm^2^ and the laser repetition rate is 200 kHz. Figure 9 presents SEM images of grooves fabricated on spherical surface under different rotation speeds. It is seen from Figure 9 that a uniform groove can be obtained for each rotation speed, given the utilized low laser energy density. Furthermore, the width of groove is independent on the rotation speed. However, a higher rotation speed leads to less pronounced HAZ with better surface quality. Therefore, a speed of 20°/s is selected for both A-axis and C-axis.

### 3.3. MALM of Continuous Patterns on Spherical Surface

With the motion path of workpiece developed in Section 3.1 and the sampled laser processing parameters obtained in Section 3.2, MALM of continious patterns on stainless steel spherical surface is carried out. Note that only the A-axis and C-axis are needed to complete the MALM-based continuous patterning of spherical surface.

**Figure 10:**
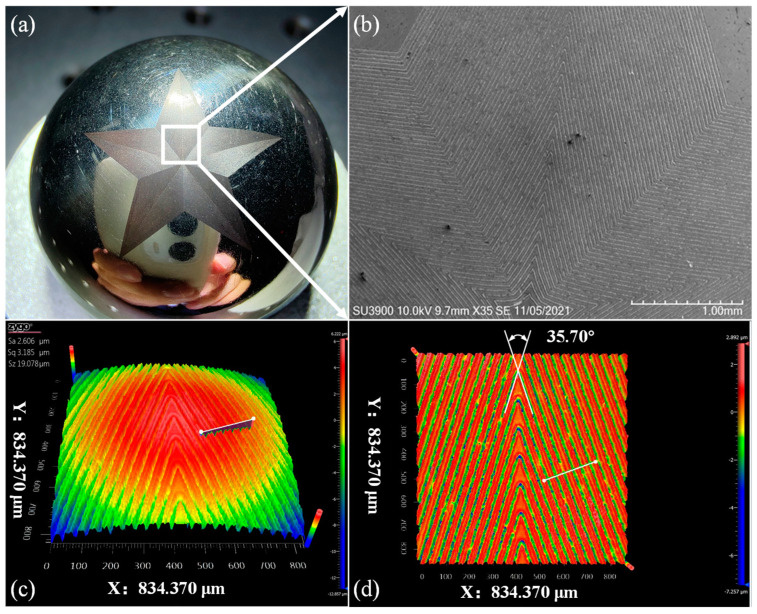
Results of MLAM of pentagonal pattern on stainless steel sphere: (**a**) Global image of the pattern; (**b**) SEM image of magnified ablation area; (**c**) spherical and (**d**) flattened surface morphology characterized by white light interferometer.

Figure 10a presents the image of fabricated pentagonal pattern on the spherical surface by a phone camera, which shows that the pentagonal shape is regular and symmetrical, and the boundaries between each part are clearly visible. Figure 10b presents the SEM image of the localized area, highlighted by the white rectangle shown in Figure 10a. The ablated array grooves on the spherical surface are finely and uniformly distributed on curved surface, indicating that the laser incident angle with respect to ablated surface normal is consistent during the laser milling process.

The machined surface morphology of the pentagonal pattern on spherical surface is also characterized by a white light interferometer. Note that although the width of each groove is tens of micrometers, the atomic force microscope with higher characterization resolution than the white light interferometer is not sufficient to measure simultaneously two grooves, due to the small measurement range of tens of micrometer. Figure 10c presents the measurement result of the local curved area. Figure 10d presents the corresponding flattened area by fitting the curved surface in Figure 10c, in which the corner angle of the pentagonal pattern can be measured. The measured corner angle of the non-planar pentagonal pattern is about 35.70°, the deviation of which from the designed value of 34.57° is 3.27%, indicating the high accuracy of MALM.

**Figure 11:**
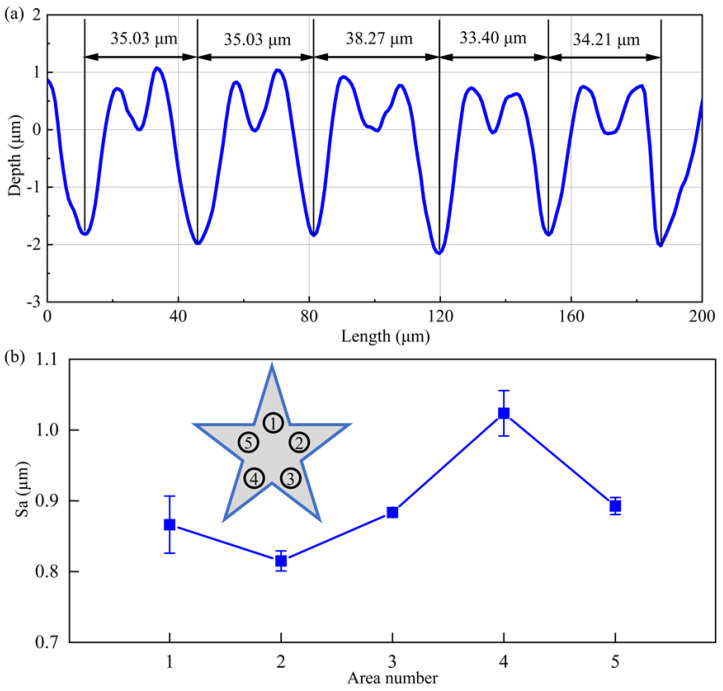
(**a**) Cross-sectional profile of fabricated grooves. (**b**) Surface roughness of five areas in the faricated pentagonal pattern.

Figure 11a further presents the cross-sectional profile of five neighboring grooves characterized by white light interferometer. In the present work the galvo scanner is not used in the processing process, and the moving speed of the laser-surface interaction point only depends on the moving speed of the platform, which is much lower than the laser moving speed when the galvanometer is used, resulting in a large number of laser pulses acting on the surface area per unit time. Since the laser beam energy presents Gaussian spatial and temporal distributions, the higher energy close to the center leads to higher vaporization velocity in the center of the groove than that at the edge. Therefore, the groove section presents a deep V-shape. There is a 1-µm high accumulation of molten materials on both sides of the processed groove edge, which is caused by the sputtering of materials on both sides of the groove with low laser energy deposited. With the measurement of five neighboring grooves, the average width of fabricated groove is 35.19 μm, which has a deviation of 12% from the designed value of 40 μm. The groove depth fluctuates around 1.94 μm, and the difference between the minimum and maximum ablation depth is 0.33 µm, indicating that the consistency of laser milling depth is well maintained for the non-planar surface texturing. It is also demonstrated that the equal arc length projection algorithm is effective in reducing the shape distortion of the processed pattern. Figure 11b further presents surface roughness measured from five locations shown in the inset, which has a maximum of 1.02 µm and a minimum of 0.81 µm. Above results demonstrate the capability of the proposed MALM in continuously fabricating a complex pentagonal pattern with uniform groove arrays and high precision of individual groove on the stainless steel spherical surface.

The height of patterns processed by the segmentation or layer slicing has a sudden change of about 8~20 μm at the boundary of segmented or sliced regions [23,24], as those strategies adjust the position of the laser or the workpiece through the jog of multi-degree-of-freedom motion platform, thus leading to discontinuous LST accompanied with the formation of clear boundaries between segmented areas or sliced girdles. However, above results demonstrate that the pentagonal patterns processed by the multi-axis linkage laser milling has a rather smaller depth fluctuation of less than 0.5 μm, and the composed grooves are continuous on the whole curved surface with the absence of any clear boundaries, due to the continuous adjusting of the relative pose between the laser and the workpiece through the linkage of each axis of the multi-axis motion platform during the machining process.

**Figure 12:**
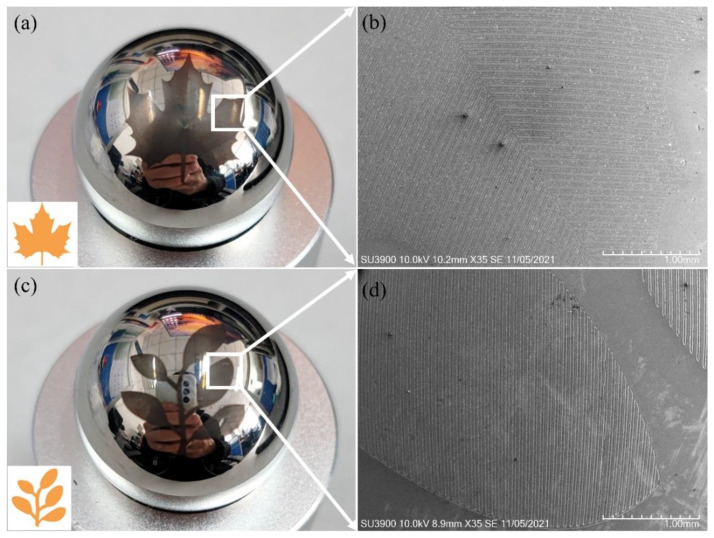
Patterns of (**a**) maple leaf and (**c**) saplings fabricated on stainless steel sphere. SEM image of local area composed of groove arrays in the fabricated (**b**) maple leaf and (**d**) saplings.

In order to further demonstrate the universality and flexibility of the proposed MALM method for non-plalar surface texturing, other two complex patterns of maple leaf and saplings are also fabricated on the stainless steel spheres using the same processing strategy and parameters. Figure 12a,c present the image of the maple leaf and saplings patterns fabricated on the stainless steel spheres, respectively. Figure 12b,d further present corresponding SEM image of the localized area highlighted by the white rectangle as shown in Figure 12a,c, respectively. It is seen from Figure 12 that continuous features of the global patterns composed of clear and uniform groove arrays are obtained on the spherical surface.

## 4. Conclusions

In the present work, we demonstrate the fabrication of complex non-planar patterns with uniform global feature and high local quality on stainless spherical surface, by utilizing a proposed method of five-axis nanosecond laser milling. Specifically, an algorithm of simultaneously controlling both the position and the posture of laser-surface interaction point is proposed to maintain the constant coincidence of laser beam with the ablated surface normal. The motion path of laser-surface interaction point is derived by the projection of planar surface pattern onto non-planar surface following the equal arc length projection algorithm, which is validated by a corresponding virtual simulation environment of MLAM. The optimal laser energy density of 2.62 J/cm^2^ and laser scanning speed of 20°/s are selected for the non-planar laser ablation. Complex pentagonal, maple leaf, and saplings patterns are successfully fabricated on stainless steel sphere, with long-range uniform and continuous groove arrays and local high precision of individual grooves. The angle error and the processing depth fluctuation for the as-fabricated pentagonal pattern are less than 5% and 0.5 μm, respectively.

## Figures and Tables

**Table 1:** Specifications of five-axis motion platform.

Specification	Value
Positioning accuracy of X, Y axis	5 μm
Travel range of X, Y axis	50 mm
Positioning accuracy of Z axis	2.5 μm
Travel range of Z axis	30 mm
Rotational accuracy of A, C axis	18″
Rotational range of A, C axis	360°
Maximum rotation speed of A, C axis	20°/s
